# Editorial: Chromosome architecture and DNA topology in prokaryotes

**DOI:** 10.3389/fmicb.2023.1355036

**Published:** 2024-01-11

**Authors:** Tamara Basta, Estelle Crozat, Ian Grainge

**Affiliations:** ^1^Université Paris-Saclay, CEA, CNRS, Institute for Integrative Biology of the Cell (I2BC), Gif-sur-Yvette, France; ^2^Laboratoire de Microbiologie et Génétique Moléculaires (LMGM), Centre de Biologie Intégrative (CBI), CNRS, Université de Toulouse, Université Toulouse III - Paul Sabatier, Toulouse, France; ^3^School of Environmental and Life Sciences, College of Engineering, Science and Environment, The University of Newcastle, Callaghan, NSW, Australia

**Keywords:** bacteria, archaea, DNA topology, DNA condensation, DNA topoisomerases, histones, NAP, SMC

DNA supercoiling and chromosome organization are at the heart of the essential cellular processes of transcription, replication, and segregation. In prokaryotes, rapid variations of DNA supercoiling are used to adapt to new conditions on a short-term basis, like changes in nutrients, stress, or a host infection, but also on a long-term basis to be fully adapted to their environment. With the advent of high throughput functional genomics assays, it became apparent that chromosomes of prokaryotes fold into higher order three-dimensional structures. How these structures are determined and regulated and what is their functional significance is a very active field of research. These processes require the action of different classes of proteins, which have all been covered by five original articles selected for this Research Topic.

First, the main enzymes that can actively modulate DNA topology are the topoisomerases, which can cut either one or both strands of DNA to modulate the supercoiling in the molecule. Topo IV is a type II topoisomerase found in all bacteria with a crucial role in decatenating both chromosomes and plasmids after their replication, as well as unlinking the pre-catenanes that are formed behind the replication forks. The precise activity of Topo IV has been difficult to assess *in vivo* as it carries out an essential process and acts over the entire chromosome. In their article, Sutormin et al. take advantage of the state-of-the-art Topo-seq technique to precisely define where Topo IV cuts in the *Escherichia coli* genome. This technique is able to quantify Topo IV activity along the chromosome by mapping the cutting sites of this topoisomerase with single nucleotide resolution. Analysis of cleavage sites defined a Topo IV recognition motif not observed previously and allowed comparison of the relative activity within the different macrodomains. Intriguingly, the authors also found evidence that Topo IV may participate, along with gyrase, in the relaxation of positive supercoils ahead of highly expressed genes, suggesting an additional function of Topo IV beyond its main role in decatenation.

The second type of proteins examined are the nucleoid associated proteins (NAP), which have long been seen as the equivalent of histones of eukaryotes and share the same double-role of DNA organization and regulation of transcription. On this front, Joyeux describes a detailed simulation, which clarifies how NAPs and cytoplasmic crowders can compact the nucleoid while allowing the genome to be expressed and segregated. Two types of proteins are considered and are seen to play complementary roles: the first are DNA binding proteins that can either self-associate and bridge two segments of DNA vs. ones that do not, and secondly “crowders” which exclude DNA from a protein mass, effectively reducing the local volume available for the DNA, therefore increasing its local concentration. These proteins can cooperate to compact DNA, but the self-associating ones do so more efficiently at lower protein concentrations.

In an experimental approach to address a similar question, Erkelens et al. focus on archaeal histones HmfA and HmfB and provide us with a biochemical/biophysical study of the DNA binding by each of the proteins. They demonstrate two distinct binding modes, one where tetramers form and are stable, single entities requiring a consensus DNA binding sequence, and a more open conformation which can polymerize to form a “hypernucleosome” and is not DNA sequence specific. These differences may be used in archaea both for general DNA compaction and for precise histone positioning affecting transcription.

The third main type of topology actors are the last that have come to light: the structural maintenance of chromosomes (SMC). In *E. coli*, the SMC complex is MukBEF, which is known to interact with Topo IV, and is seen to be associated preferentially close to the replication origin. Japaridze et al. use a MreB inhibitor to generate large and round *E. coli* cells. This technique allows them to visualize a donut-shaped nucleoid, along with fluorescent origin and terminus foci, in *matP-* or mutant *mukBEF* background. They show that MatP, the *ter*-binding domain protein, inhibits chromosome compaction by MukBEF within the *ter* region.

Finally, Camus et al. shed light on a SMC-like protein, RecN, which in bacteria is known for its role in the repair of double strand breaks by homologous recombination. Recent data, however, points to the role of RecN in other genetically distinct DNA repair pathways. The authors investigate this by looking into the RecN-driven nucleoid management and sister chromatid dynamics in *E. coli* in response to two different genotoxic drugs. They report the involvement of RecN in the repair of different types of lesions, thus identifying this SMC-like protein as one of pivotal elements in handling genotoxic stress in bacteria with larger implications for understanding how these organisms survive in hostile environments.

This Research Topic is nicely completed by two reviews. Junier et al. provide an extensive summary of the current understanding of DNA supercoiling in bacteria, how topoisomerases regulate this supercoiling, the computational methods to model DNA topology based on physical laws and concepts, and how this affects the macromolecular architecture of the chromosome. The authors discuss the current prevalent mathematical models in terms of relevance, predictive capacity, and limitations with particular emphasis on the relationship between supercoiling and gene transcription, DNA replication, and genome folding. The review is intended for both biologists and physicists with particular emphasis on keeping the language accessible to both communities to foster future interactions.

The second review examines how replication is terminated (Goodall et al.) and why the terminus region has a precise and conserved architecture. In bacteria, replication termination systems limit where replication forks meet to a narrow zone and reduce the deleterious outcomes that may result from these fork fusion events. The replication termination proteins along with RecG, other recombinases, and 3′ exonucleases work together to ensure that two converging replication forks terminate properly; in their absence, improper DNA structures or over-replication can occur.

Overall, this Research Topic highlights the importance of DNA topology, supercoiling, and chromosome organization to ensure the proper functioning of the cell.

## Author contributions

TB: Writing – original draft, Writing – review & editing. EC: Writing – original draft, Writing – review & editing. IG: Writing – original draft, Writing – review & editing.

